# Expression of heat-shock protein 72 mRNA in relation to heart rate variability of Sahiwal and Karan-Fries in different temperature-humidity indices

**DOI:** 10.14202/vetworld.2016.1051-1055

**Published:** 2016-10-07

**Authors:** Prava Mayengbam, T. C. Tolenkhomba, R. C. Upadhyay

**Affiliations:** Dairy Cattle Physiology Division, National Dairy Research Institute, ICAR, Karnal - 132 001, Haryana, India

**Keywords:** heat-shock protein 72 mRNA, Karan-Fries, Karnal, Sahiwal, stress, temperature-humidity index

## Abstract

**Aim:**

To investigate the effect of temperature-humidity index (THI) on the expression pattern of heat-shock protein 72 (HSP72) mRNA of Sahiwal and Karan-Fries (KF) cattle in different THIs.

**Materials and Methods:**

Five different periods of a year were selected based on combinations of T_max_/T_min_, viz., P1: <20°C/<10°C; P2: >20°C/<10°C, P3: <30°C/<15°C; P4: >35°C/<20°C, and P5: >35°C/>20°C. The THI was calculated from the records of temperature and relative humidity in different periods. Heart rate variability (HRV) was calculated from electrocardiogram records in different periods. HSP72 mRNA expression was estimated by reverse transcription polymerase chain reaction.

**Results:**

The THI recorded during P1, P2, P3, P4, and P5 were 55.5, 60.3, 70.1, 74.5, and 79.0, respectively. THI in P4 and P5 were stressful to animals. HSP72 mRNA expression increased during cold stress in P1 in Sahiwal and heat stress in P4 and P5 in both Sahiwal and KF. Sahiwal maintained increased HSP72 mRNA expression longer than KF without causing a significant change in HRV.

**Conclusion:**

Both low THI in winter and high THI in summer increased HSP72 mRNA of Sahiwal and KF without significant change in HRV. Thermotolerance of Sahiwal could be due to the maintenance of higher HSP72 expression longer than KF in prolonged heat stress in summer.

## Introduction

Thermal stresses trigger a complex program of gene expression and biochemical adaptive responses. Heat-shock protein (HSP) 72, a member of the HSP70 family, is a highly stress-inducible protein. HSP72 transcription is increased by heat shock as well as other stress stimuli and can be an indicator of stress in cells [1]. Stress-induced synthesis of HSPs represents a generalized molecular mechanism displayed by almost all cells, but individual animals differ in their capacity to manage with stress [2]. It has also been reported that HSP72 is induced when lymphocytes were subjected to heat stress *in-vitro* in cattle [3,4] and buffaloes [5]. Studies in natural climatic conditions and in psychrometric chamber in Sahiwal and Karan-Fries (KF) heifers revealed different expression patterns of HSP72 [6,7].

Temperature-humidity index (THI) had been indicated to be a good measure in the assessment of heat stress in livestock [8]. Expression of Mn-superoxide dismutase (SOD) and Cu-SOD mRNA of Sahiwal and KF were found to be different in different THIs [9]. HSP72 gene expression of Surti buffalo heifers was different in different THIs [10].

There is, however, no sufficient literature available in respect of HSP72 mRNA expression of Zebu cattle Sahiwal and crossbred KF in relation to their physiological response in different THIs. The study will add in a better understanding of the cellular responses involved in the better thermotolerance of Zebu and its crossbreds as compared to other cattle breeds of exotic origin.

## Materials and Methods

### Ethical approval

The present investigation was carried out after the approval of the Institutional Animal Ethics Committee.

### Animals and blood sampling

Sahiwal and KF heifers numbering 12 each were selected from the herd maintained at the National Dairy Research Institute, Karnal, India. The animals were in the age group of 2-2.5 years, and average body weight was 301.3±6.91 kg. The animals were given a maintenance concentrate mixture at 1 kg/animal in addition to *ad libitum* roughages and water as per Kearl [11]. Concentrate mixture consisted of mustard cake, maize, wheat bran, rice bran, mineral mixture, and salt. The crude protein and total digestible nutrient of diet were 12% and 60%, respectively. 2 ml each of blood samples was collected for separation of peripheral blood mononuclear cells (PBMCs) and subsequent isolation of total RNA.

### Record of THI

Five different combinations of T_max_ and T_min_ which were selected based on the climograph prepared from the records of previous 10 years. Five different T_max_/T_min_ conditions were P1: <20°C/<10°C; P2: >20°C/<10°C; P3: >30°C/<15°C; P4: >35°C/<20°C, and P5: >35°C/>20°C. The different periods, *viz*., P1, P2, P3, P4, and P5 of study were during the periods from 26^th^ December, 2007, to 7^nd^ January, 2008; 7^th^ December to 13^th^ December, 2007; 16^th^ October to 31^st^ October, 2007; 7^th^ April to 23^rd^ April, 2008, and 24^th^ May to 7^th^ June, 2008, respectively. Prevailing temperature at Karnal as recorded at the Central Soil Salinity Research Institute, Karnal, was obtained. Everyday records of minimum/maximum ambient temperature, dry bulb, and wet bulb temperature were recorded at I: 0722\0830 and II: 1422 h IST. The THI was calculated as per the formula given by the National Research Council [12].

THI=0.72(T_db_+T_wb_)+40.6

Where, T_db_ and T_wb_ were the dry bulb and wet bulb temperatures, respectively.

### Record of heart rate (HR) variability (HRV)

The electrocardiograms (ECGs) of each of the animals were recorded using bipolar limb leads of student physiograph. The vertical sensitivity was adjusted to give 10 mm deflections per mV, and the paper speed was kept at 25 mm/s. The HRV of individual animals was calculated from the ECGs of individual animals. The time domain analysis [13] was used to find out HRV. The standard deviation of the normal-to-normal (SDNN) R-R intervals; ms and root mean square of successive differences of R-R intervals; ms (RMSSD) were calculated from continuous record of 5 min to find out HRV. ECG was recorded before blood collection that was in between 06:00 h IST and 07:00 IST.

### Analysis of blood samples

PBMCs were separated by gradient centrifugation with Histopaque (Sigma). The PBMC was cultured by incubation at 38°C for 3 h in lymphocyte-selective media, Roswell Park Memorial Institute-1640 supplemented with penicillin and streptomycin at 100 µg/ml and bovine serum at 0.5% to obtain the lymphocytes. The lymphocytes were collected by centrifugation and dried to make cell pellets. The cell pellets were stored at −80°C until RNA isolation.

Total RNA was isolated using Tri-reagent (Sigma) as per the guidelines with slight modifications. After determining the purity and quality of RNA, the RNA samples were reversed transcribed to cDNA using RevertAid First Strand cDNA synthesis kit (Fermentas). From the reversed transcribed cDNA, polymerase chain reaction (PCR) was carried out for HSP72 gene using specific primers. The glyceraldehyde 3-phosphate dehydrogenase (GAPDH) gene was used as a housekeeping gene for relative measure of expression of HSP72. The details of the primers used in the experiment were presented in Table-1.

**Table-1:** Details of HSP72 and GAPDH genes.

Gene and primer sequence	T^A^ (°C)	Amplicon size (bp)	Gene bank accession number (Reference)
HSP872 5’-AACATGAsAGAGCGCCsGTGGAGG-3’ 5’-GTTACACACCTGCTCCAGCTCC-3’	63	169	U02892 [4]
GAPDH 5’-CCCATCACCATCTTCCAGG-3’ 5’-AGTGAGCTTCCCGTTCAGC-3’	63	471	[14]

HSP72=Heat-shock protein 72, GAPDH=Glyceraldehyde 3-phosphate dehydrogenase

The PCR of HSP72 and subsequent record of amplified products by sodium dodecyl sulfate polyacrylamide gel electrophoresis were carried out by following the methods as described by Sambrook *et al*. [15] with slight modifications. The PCR reaction mixture (25 µl) consisted of 2.5 µl ×10 Tag polymerase buffer, 6 µl 25 mM MgCl_2_, 0.5 µl 10 mM deoxynucleotide triphosphate, 0.5 µl 10 mM forward primer, 0.5 µl 10 mM reverse primer, 2 µl cDNA, 0.15 µl 5 U/µl Taq polymerase, and 12.85 µl nuclease free water. The amplified products of PCR were electrophoresed in agarose gel containing ethidium bromide (5 µg/100 ml gel). Semi-quantitative measure of expression of mRNA of genes of interest was obtained from the digital pictures recorded from the electrophoresed products. The integrated density value (IDV) of each of the bands was measured in GelDoc (Image AIDE 10990*Syn1234*mpcs5870337c Spectronics, Gel Doc Software). Expression of mRNA of the gene of interest was obtained from the ratio of IDV of specific gene to IDV of GAPDH.

The data were analyzed using SYSTAT Version 6.0.1, Copyright^©^ 1996, SPSS Inc., Chicago, IL, USA. The one-way analysis of variance was carried to find out the effect of periods and breed. Fisher’s least-significant-difference test was applied to find out matrix of pair-wise comparison probabilities between different groups.

## Results

### THI

The variation in prevailing T_max_/T_min_, T_db_, T_wb_, and the measure of THI recorded during the period of study was presented in Table-2. The average THIs in P1, P2, P3, P4, and P5 were 55.5, 60.3, 70.1, 74.5, and 79.0, respectively.

**Table-2:** Ambient temperature and THI during different periods.

Parameters	Periods				

	P1	P2	P3	P4	P5
T (°C)					
Max	19.0	21.4	31.3	35.5	35.6
Min	4.3	8.7	14.5	17.4	22.8
Average	11.6	15.1	22.9	26.5	29.2
THI					
Max	63.4	66.3	77.4	81.4	82.6
Min	48.6	54.0	63.0	68.0	75.5
Average	55.5	60.3	70.1	74.5	79.0

THI=Temperature-humidity index

### HRV

HRV as calculated from the ECG records of the animals was presented in Table-3. The SDNN (ms) and RMSSD (ms) calculated from the ECG revealed no significant differences between the breeds in any of periods of the study. HRV of Sahiwal and KF were neither influenced by THI nor by the cold temperature of winter.

**Table-3:** SDNN (Mean±SEM) and RMSSD (Mean±SEM) of Sahiwal and KF during different periods of T_max_/T_min_ combinations.

HRV	Breed	Periods				

		P1	P2	P3	P4	P5
SDNN (ms)	Sahiwal	57.87±12.29	58.30±8.02	71.36±6.82	42.64±6.56	33.49±6.11
	KF	53.78±15.69	38.12±6.27	45.73±s7.85	52.57±12.94	40.63±11.93
RMSSD (ms)	Sahiwal	36.50±5.50	36.49±5.75	65.80±9.94	28.52±6.00	25.45±4.79
	KF	54.45±25.08	26.65±3.58	30.83±5.93	46.07±16.00	35.23±14.97

SDNN=Standard deviation of the normal-to-normal, RMSSD=Root mean square of successive differences, KF=Karan-Fries, SEM=Standard error of mean, HRV=Heart rate variability

## Effect of THI on HSP72 mRNA expression

**Figure-1:**
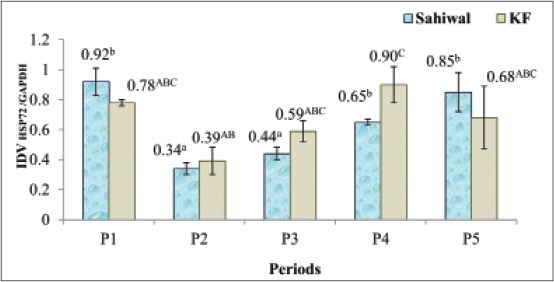
Effect of temperature-humidity index on heat-shock protein 72 mRNA expression of Sahiwal and Karan-Fries. Bars indicate means with standard error. Means of the same breed with different superscripts differ significantly (p<0.05).

The effect of different THIs on the expression of HSP72 mRNA was evident in both the breeds (p<0.05) as presented in Figure-1. HSP72 expression in Sahiwal increased due to cold stress in PI as compared to P2 and P3 (p<0.05). The THI ≥74.5 recorded in P4 (T_max_/T_min_ - 36.0°C/17.8.0°C) and P5 (T_max_/T_min_ - 37.79°C/23.16°C) increased HSP72 mRNA expression higher than that recorded in P1 (THI=55.5) and P3 (THI=60.3). KF had almost stable HSP72 mRNA in winter and THI up to 62.8. THI of 74.5 increased HSP mRNA in KF significantly higher (p<0.05) than in P2 (T_max_/T_min_ - 21.4°C/8.7°C). There were no significant differences between the breeds in HSP72 expression.

## Discussion

The T_max_/T_min_ in different periods fell in the ranges as per the climograph of the previous 10 years. In intensive animal housing with environmental modification, temperature alone is also the usual control parameter. However, by combining temperature and humidity effects, the THI does capture much of the impact of warm to hot thermal environments on animals [8]. The THI is commonly used as an indicator of the intensity of climatic stress on animals, where THI of 72 and below is considered as no heat stress, 73-77 as mild heat stress, 78-89 as moderate, and above 90 as severe [16]. As per the record of THI in different T_max_/T_min_ conditions in the present study (Table-2), the P1, P2, and P3 were not stressful to the animals and P4 and P5 were stressful for the animals.

The measurement of HRV had been indicated to be a noninvasive approach to measure stress in calves and cows [17,18]. HRV analysis has been indicated to be a reliable method in the assessment of different types of stress [19-21]. To find out the response of indigenous and crossbred cattle to cold and heat stress, further investigation was carried out to find out HRV which is an instantaneous measure of sympathetic and parasympathetic activity in animal’s body. The variations in HR due to heat or cold stress were well within the physiological adjustments in both the breeds. Maintenance of a stable HRV by both the breeds indicated similar thermotolerance between Sahiwal and KF in terms of its physiological indices. It could be possible that after several generations of thermal exposures in extreme temperature that prevailed during the summer at Karnal might have led to the better thermal adaptability of KF.

To find out a cellular process which could be a marker for assessment of stress in Sahiwal and KF, further investigation was carried out to measure HSP72 mRNA expression in both the breeds. The effect of different THIs on the expression of HSP72 mRNA was evident in both the breeds (p<0.05) similar to previous reports in lactating cows [22]. Although the THI recorded during P1 was comfortable for cattle, Sahiwal experienced a mild degree of cold stress during winter (P1: T_max_/T_min_ - 16.7°C/2.5°C) which was evident with higher HSP72 expression (p<0.05) as compared to P2 (T_max_/T_min_ - 20.4°C/9.0°C) and P3 (T_max_/T_min_ - 30.8°C/13.6°C) similar to heat-adapted goats which had higher HSP70 expression in cold stress [23]. It could suggest that only the measure of THI was not sufficient to detect the presence of stress especially when there was cold stress. The presence of higher HSP72 expression in Sahiwal due to cold and heat stress and higher HSP72 expression in KF due to heat stress resembled the finding of higher HSP70 during summer in both heat- and cold-adapted goats [23] and acute heat stress-induced HSP70 expression in Surti buffalo heifers [10].

HSP72 is regarded as an inducible protein that is normally expressed at low levels under nonstress conditions and at markedly higher levels after exposure to stimuli, such as stress, infections, or hormonal stimulation [24-26]. When HSP72 protein was estimated in natural climatic conditions and in thermal exposure in psychrometric chamber, Sahiwal heifers were found to have higher ranges of protein than KF heifers [6,7]. When the kinetics of expression of HSP72 was investigated, differential expression patterns were recorded in between buffalo and cattle. Chronic heat exposure of PBMC *in-vitro* caused higher HSP72 expression in PBMC of cattle than the buffaloes [27]. Such differences could be attributed to the different capabilities of thermotolerant breed Sahiwal and its crossbred KF. Studies in thermal stress acclimatized and nonacclimatized rats revealed that in initial phase of heat acclimation augmented increase in HSP72 transcription was not accompanied by HSP synthesis and the inability to elevate HSP cytosolic level ruled out their direct cryoprotective role [28]. HSP72 mRNA was found to be higher in high milk yielders than in low milk yielder Sahiwal and KF cows [29]. Maintenance of higher HSP72 mRNA in KF in different THIs in the present study with lower ranges of HSP72 in KF in previous reports [6,7] indicated the better-equipped mechanism of HSP72 transcription and HSP translation in Sahiwal than in KF. The present finding also supports the findings that camels had higher HSP72 in the fibroblasts to withstand the exposures to extreme temperatures [30].

In the absence of any detectable changes in the spontaneous record of HRV, the measurement of varying levels of expression of HSP72 mRNA in different THIs indicated HSP72 as a potent marker for cold and heat stress. The better machinery in transcription and subsequent translation to HSP72 in the cattle breeds originated and developed in India could be the basis for having good thermal adaptability in natural climatic conditions of the country.

## Conclusion

Thermal stress includes both heat stress, during extreme summer as well as cold stress, during extreme winter. The effect of high temperature is further aggravated when it is accompanied by high ambient humidity. The physiological responses of Zebu cattle, Sahiwal, and a crossbred of Sahiwal and KF in terms of its HRV were almost similar in different THIs showing similar levels of heat tolerances. Although the HRV was stable in cold and heat stress, the measure of HSP72 mRNA of Sahiwal and KF in different THIs both in winter and summer detected a mild degree of cold stress experienced by Sahiwal and heat stress experienced by both Sahiwal and KF in natural climatic conditions. The difference in the expression pattern of HSP72 by two breeds explains the genetic differences of Sahiwal and KF in terms of their respective thermotolerance.

## Authors’ Contributions

PM and TCT performed the experiments and analyzed the results statistically. RCU designed the experiments, and the experiments were carried out under the supervision of RCU.
